# Opioids Regulate the Immune System: Focusing on Macrophages and Their Organelles

**DOI:** 10.3389/fphar.2021.814241

**Published:** 2022-01-12

**Authors:** Shaohua Wen, Yuan Jiang, Shuang Liang, Zhigang Cheng, Xiaoyan Zhu, Qulian Guo

**Affiliations:** ^1^ Department of Anesthesiology, Xiangya Hospital, Central South University, Changsha, Hunan, China; ^2^ National Clinical Research Center for Geriatric Disorders, Xiangya Hospital, Central South University, Changsha, China

**Keywords:** opioids, macrophages, tumor progression, human immunodeficiency virus, organelles

## Abstract

Opioids are the most widely used analgesics and therefore have often been the focus of pharmacological research. Macrophages are the most plastic cells in the hematopoietic system. They show great functional diversity in various organism tissues and are an important consideration for the study of phagocytosis, cellular immunity, and molecular immunology. The expression of opioid receptors in macrophages indicates that opioid drugs act on macrophages and regulate their functions. This article reviewed the collection of research on effects of opioids on macrophage function. Studies show that opioids, both endogenous and exogenous, can affect the function of macrophages, effecting their proliferation, chemotaxis, transport, phagocytosis, expression of cytokines and chemokine receptors, synthesis and secretion of cytokines, polarization, and apoptosis. Many of these effects are closely associated with mitochondrial function and functions of other organelles in macrophages. Therefore, in depth research into effects of opioids on macrophage organelles may lead to some interesting new discoveries. In view of the important role of macrophages in HIV infection and tumor progression, this review also discusses effects of opioids on macrophages in these two pathological conditions.

## Introduction

Opioids, such as morphine, are widely used in clinical pain treatment worldwide and are most commonly used to treat pain from cancer and various forms of acute intraoperative pain. However, opioids pose a risk to patients and society, as their misuse or abuse can lead to addiction and overdose (Webster, 2017). In recent years, the number of people who have died from opioid overdose has increased worldwide, exposing a potential opioid drug crisis (Volkow and Blanco, 2021).

Macrophages are immune cells that are studied for their role in cellular phagocytosis, cellular immunity, and molecular immunology. They play a central role in regulating humoral and cellular immunity against infectious diseases and cancer. Macrophages can eradicate bacteria invading the body; phagocytize foreign particles, aging or damaged cells and the degenerating intercellular matrix; kill tumor cells; and activate lymphocytes or other immune cells.

The immunomodulatory effects of opioids in the context of immunosuppression and infection have been widely reviewed (Liang et al., 2016; Boland and Pockley, 2018; Plein and Rittner, 2018). The review published by Eisenstein in 1998 elaborated on the influence of opioids on macrophage function and the regulation of immune response (Eisenstein and Hilburger, 1998). Many studies have shown that human and rodent macrophages can express opioid receptors. Opioid drugs, such as morphine, interacting with opioid receptors can inhibit phagocytosis and chemotaxis of macrophages. *In vitro* experiments have also shown that opioids or opioid peptides have direct effects on macrophages.

This review paper summarized the latest evidence regarding effects of opioids on macrophage function in the last 20 years. Morphine is the most well studied opioid with regards to macrophage function and significantly less research has been done on other opioids, such as fentanyl, methadone, tramadol, oxycodone, hydromorphone, and buprenorphine. Opioids, both endogenous and exogenous, can have an effect on the function of macrophages, including macrophage proliferation, chemotaxis, transport, phagocytosis, expression of cytokines and chemokine receptors, synthesis and secretion of cytokines, polarization, and apoptosis. These effects have been mainly evaluated *in vitro*, and the results are often contradictory, depending on different experimental conditions, for example cultured cell type, cell line or clone, culture duration, culture medium composition, dose, and opioid exposure time. In the following sections we introduce the opioids, opioid receptors, and macrophages, and discuss their relationship. We also discuss the relationship between opioids and organelles in macrophages, a promising topic for future studies. Additionally, in view of the critical role of macrophages in HIV infection and tumor progression, we discuss the influence of opioids on macrophages under these two pathological conditions.

## Classification and Basic Pharmacology of Opioids

Opioids can be divided into natural opioid alkaloids, semi-synthetic opioids, and synthetic opioids, depending on the source of the compound and the processing involved. Four natural alkaloids—morphine, codeine, papaverine, and thebaine—are all extracted from *Papaver somniferum*, among which, morphine is the most widely used in the clinic. Semi-synthetic opioids, such as diamorphine and oxycodone, are produced by simple chemical treatment of natural opioid alkaloids. Synthetic opioids, widely used in clinics in the past half-century, can be further subdivided into four groups: morphinan derivatives, diphenyl heptane derivatives, benzomorphan derivatives, and phenylpiperidine derivatives. Opioids can also be classified as agonists, partial agonists, antagonists, and agonist-antagonists, depending on their effect on opioid receptors.

Since the last century, the classification of opioid receptors has changed with time. At present, the commonly used NC-IUPHAR divides opioid receptors into four categories: DOP or δ, KOP or κ, MOP or μ, and nociceptin receptor (NOP) (James and Williams, 2020). The first three are classical opioid receptors, all of which are G protein-coupled receptors (GPCRs). The NOP receptor is also a GPCR system, which has obvious similarity with the known amino acid sequence of classical opioid receptors. NC-IUPHAR states that the NOP receptor is a non-opioid branch of the opioid receptor family because when the NOP receptor is activated by an agonist at the cellular level, it produces similar effects to the classic opioid receptors mentioned above. Classical opioid receptors have been divided into subtypes, µ_1_, µ_2_, and µ_3_ for MOP, δ_1_ and δ_2_ for DOP, and κ_1a_, κ_1b_, κ_2a_, κ_2b_ and κ_3_ for KOP.

Previous studies have confirmed that the primary mechanism of opioid-induced analgesia is through activation of the midbrain MOP receptor in the central nervous system. MOP agonists have an analgesic effect by indirectly increasing neuronal flow in the nucleus reticularis paragigantocellularis and periaqueductal gray descending pathway, or by directly inhibiting peripheral nociceptive afferent sensitivity. In addition, MOP agonists can indirectly inhibit the transmission of spinal cord pain and reduce the sensation of spinal cord injury. Although analgesic properties of opioids may be attributed to the activation of the MOP receptor, this may also be the reason for many of the side effects associated with opioids. Opioids may lead to euphoria and decreased awareness, making them prone to abuse. They also affect the respiratory system, reducing the respiratory rate and airway reflex, which is considered to be beneficial during anesthesia. Although opioids are generally considered to maintain the stability of the heart, the release of histamine and the decrease of systemic vascular resistance and blood pressure are evident in the case of morphine. They can also cause constipation, nausea, vomiting, urinary retention, itching, muscle stiffness, mydriasis, and irritability in some individuals. Many side effects may limit their use.

## Overview of Macrophages

In adult mammals, macrophages are not only present in the blood, but also in various tissues throughout the body. They demonstrate huge anatomical and functional diversity in these tissues. Depending on the location, the names and shapes of macrophages may also be different. For example, they are referred to as pulmonary macrophages in the lungs, microglia in the nervous system, Kupffer cells in the liver, and osteoclasts in the bone.

In the past, various systems were used to classify phagocytic mononuclear cells and their precursors, but the most successful is the mononuclear phagocytic system, which is defined as progenitor cells in the bone marrow, monocytes in the blood, and macrophages in tissues as phagocytic mononuclear cells or precursors. Another classification method reflects two extreme conditions of macrophages, including activated and alternative activated macrophages, otherwise known as M1 and M2, respectively. Under the influence of the local microenvironment cytokines, macrophage populations usually differentiate into two phenotypes, in a process known as macrophage polarization (Wang et al., 2014; de Gaetano et al., 2016; Yunna et al., 2020). M1 macrophages participate in the positive immune response by secreting pro-inflammatory cytokines and chemokines and presenting antigens, thereby monitoring the immune system. M2 macrophages only have a weak antigen-presenting ability and play an important role in immune regulation by secreting inhibitory cytokines, such as IL-10 or TGF-β (Wynn et al., 2013). However, it is worth noting that the idea of dichotomy is largely oversimplified and is mainly based on the *in vitro* response to polarization stimulation. In fact, many macrophage subtypes have overlapping functions and phenotypes since they are exposed to hundreds of different stimuli. These functions are integrated by complex signal pathways and finally produce specific effects.

Macrophages are the most plastic cells in the hematopoietic system and show great functional diversity in various tissues of organisms. Changes in macrophage phenotype and function lead to dramatic changes in cell metabolism. These metabolic adaptations, in turn, support the activity of macrophages and maintain their polarization in specific environments (Viola et al., 2019). For example, M1 macrophage metabolism mainly depends on glycolysis. Interestingly, in M1 macrophages, two points of interruption in the tricarboxylic acid (TCA) cycle results in the accumulation of itaconate (a microbicidal compound) and succinate. The accumulation of succinate leads to the stabilization of hypoxia-inducible factor 1α (HIF1α), which in turn activates the transcription of glycolytic genes, thus maintaining the glycolytic metabolism of M1 macrophages.

Macrophages not only regulate the normal metabolic balance and immune response *in vivo*, but also play an important role in the pathophysiological process of various system diseases. Studies have confirmed that macrophages play an important role in the development of inflammatory bowel disease, atherosclerosis, diabetic nephropathy, AIDS, and tumors. The plasticity of macrophages in the process of intestinal inflammation indicates that these cells not only play an extensive role in the occurrence of inflammation, but also in termination, healing, and repair of inflammation (Moreira Lopes et al., 2020). During the development and progression of atherosclerosis, macrophages respond to various environmental signals, such as lipids and their derivatives, pro-inflammatory and anti-inflammatory cytokines, and heme in aging red blood cells, thus regulating different phenotypes of macrophages (Jinnouchi et al., 2020). In the experimental model of diabetes mellitus, it has been found that macrophage infiltration (mainly M1) occurs in the early stage of the disease, which is one of the reasons renal matrix hyperplasia and irreversible pathological changes of the glomerulus (Calle and Hotter, 2020). Tumor-associated macrophages (TAMs) are the key driving factors of tumor progression, metastasis, and resistance therapy (Wu et al., 2020).

## Macrophage-Associated Opioid Receptors

Opioids are agonists of MOP, DOP, and KOP receptors, while nociception/orphanin FQ peptide is an agonist of NOP receptors. In animal models, all four opioid receptors on neurons can induce analgesia, but the most relevant ones are MOP receptors and their agonists, such as morphine and fentanyl. Opioids can affect the function of immune cells and their role in immunosuppression and infection has been widely discussed. Here, we analyze the expression of opioid receptors in macrophages.

Four opioid receptors have been found in the human, rhesus monkey, rat, and mouse macrophages at mRNA and protein levels (Eisenstein, 2019; Machelska and Celik, 2020) (Table 1). Sedqi et al. (1995) confirmed expression of MOP receptor-related transcripts in rat peritoneal macrophages by reverse transcription-polymerase chain reaction (RT-PCR). Ignatowski et al. analyzed the differential expression of the KOP receptor on mouse lymphocytes and mouse macrophages at different maturation stages after selective induction and found that resident peritoneal macrophages showed more specific receptor markers and about 50% of resting macrophages expressed KOP receptors (Ignatowski and Bidlack, 1999).

**TABLE 1:** The distributions of opioid receptors in macrophages among different models.

Opioid receptor	mRNA, protein	Cell types	References
MOR	mRNA	Rat peritoneal macrophages	Sedqi et al. (1995), Gomez-Flores et al. (2001), Stanojević et al. (2008), Machelska and Celik (2020)
	—	Rat splenic macrophages	Gomez-Flores et al. (2001)
	—	Mice peritoneal macrophages	Balog et al. (2010)
	mRNA	Human macrophages	Machelska and Celik, (2020)
KOP	protein	Mouse peritoneal macrophages	Ignatowski and Bidlack (1999)
	protein	Rat alveolar macrophage cell line (NR8383)	Zeng et al. (2020)
DOP	—	Rat peritoneal macrophages	Gomez-Flores et al. (2001), Stanojević et al. (2008)
	—	Rat splenic macrophages	Gomez-Flores et al. (2001)
	protein	Murine macrophage cell line (RAW 264.7)	Husted et al. (2005)
	—	Rat peritoneal macrophages	Stanojević et al. (2008), Tang et al. (2011)

## Opioids Affect the Chemotaxis, Recruitment, Migration, and Phagocytosis of Macrophages

Previous studies mostly focused on the effects of morphine, at different doses and with different administration methods, on macrophage function. Limited studies have systematically analyzed the effects of morphine on macrophage recruitment and migration. These studies have shown that morphine treatment results in a significant delay and decrease in macrophage recruitment at the wound site, which was attributed to the inhibition of effective macrophage chemokine monocyte chemoattractant protein-1 (MCP-1). The immunosuppressive effect of morphine on the recruitment of early innate immune cells makes neutrophils unable to fully migrate to the injury site within 24 hours after injury. This leads to less production and secretion of MCP-1 and reduced chemotaxis gradient, which is crucial for subsequent macrophage recruitment. Macrophage infiltration decreases, resulting in decreased neutrophil clearance and increased pro-inflammatory response. Morphine regulates inflammation induced CCR2 expression, which may be a potential mechanism by which morphine affects the continuous recruitment of macrophages (Martin et al., 2010). There may also be other mechanisms by which morphine affects macrophage recruitment and migration, therefore further studies are warranted.

Phagocytosis by macrophages is extremely important. Macrophages swallow and process large foreign bodies, old waste excreted by cells and red blood cells at the end of their life. Macrophages can locate and infiltrate a site of inflammation to deal with foreign bodies. Some studies have explored the effects of opioids, such as morphine, on macrophage phagocytosis. Bhaskaran et al. found that a high dose of morphine (HDM), equal to 40 mg/kg body weight every 12 h, impaired the ability of macrophages to inhibit and kill bacteria by phagocytosis (Bhaskaran et al., 2007). In the HDM group, peritoneal bacterial leakage and the number of macrophages migrating into the peritoneal cavity decreased significantly. HDM promoted the phosphorylation of p38MAPK in macrophages, while morphine pretreatment attenuated this effect. Tomassini et al. showed that MOP and DOP receptors mediate morphine’s effect on the phagocytosis of mouse peritoneal macrophages (Tomassini et al., 2003). Ninković et al. found that long-term morphine treatment increased cAMP and activated protein kinase A in macrophages, resulting in inhibition of Rac1 GTPase and p38MAPK, weakening actin polymerization and FCGR-mediated phagocytosis, and reducing bacterial clearance (Ninković and Roy, 2012). Ninković et al. (2016) confirmed that opioid therapy can reduce the phagocytosis by macrophages of gram-positive bacteria compared to gram-negative bacteria. Lipopolysaccharide (LPS)-stimulated chronic morphine-treated macrophages could enhance phagocytosis and killing of gram-positive and gram-negative bacteria through the p38MAPK-dependent signaling pathway. These studies explored the effects of morphine on macrophage phagocytosis from different aspects (including different doses of morphine, receptors of morphine on macrophages, and types of bacteria phagocytized). However, the relevant downstream mechanism is not clear and need further exploration.

Several other studies have compared the effects of different opioids on macrophage phagocytosis. Shirzad et al. (2009) compared the effects of morphine and tramadol on the phagocytic activity of mouse peritoneal phagocytes and found that after 10 days of treatment, the number of phagocytes and the phagocytic index in the morphine-treated group decreased, while the number of phagocytes in the tramadol group increased. Acute exposure to morphine and methadone inhibited phagocytosis in human monocyte-derived macrophages (h-MDMs) in a dose-dependent manner. In contrast, long-term exposure leads to the eventual normalization of phagocytosis, indicating that a hypothetical state of tolerance to opioids has been formed. When opioids are withdrawn from long-exposed h-MDMs with tolerance to opioids and reintroduced, phagocytosis is inhibited again (Delgado-Vélez et al., 2008). Comparing the effects of different opioids on macrophage phagocytosis and exploring the mechanism of effects are helpful to regulate the immune state of the body and the clinical application of opioids.

## Opioids Affect the Production of Various Cytokines and the Polarization of Macrophages

In addition to affecting the phagocytosis of macrophages, opioids can also act on opioid receptors on macrophages, thereby affecting intracellular molecular targets and the production of various cytokines (Table 2). The change of the production ratio of various cytokines can provide a basis for judging the polarization of macrophages in different directions (M1 or M2). Therefore, in this section, we talk about the effects of opioids on the production of various cytokines and the polarization of macrophages.

**TABLE 2:** Opioids affect the molecular targets of macrophages and the production of various cytokines.

Opioids	*Vivo./vitro.*	Cell type or mouse model	Molecular	Cytokines	References
Morphine, fentanyl, methadone	*Vivo.*	Murine peritoneal macrophages	CD14, CD80, CD86, MHCⅡ	IL-6, TNF-α, IL-10, ROIs	Filipczak-Bryniarska et al. (2012)
Morphine	*Vivo.*	Murine peritoneal macrophages	Not mentioned	IL-10, IL-12	Limiroli et al. (2002)
Morphine	*Vivo.*	Murine peritoneal macrophages	Not mentioned	NO_2_^−^	Pacifici et al. (1995)
Morphine	*Vivo. and vitro.*	Murine splenic macrophages	Not mentioned	NO	Alexander et al. (2005)
Morphine	*Vivo. and vitro.*	WT and RelB−/− mice, peritoneal macrophages	NF-κB, RelB	IL-1, TNF-β, IL-12, IL-10	Martucci et al. (2007)
Oxycodone, buprenorphine	*Vivo. and vitro.*	CBA/J mice, oil-induced peritoneal Mf	Not mentioned	ROS, NO	Kozlowski et al. (2017)
Heroin	*Vivo. and vitro.*	Murine peritoneal macrophages	Not mentioned	↑IL-1 β, INF- γ, IL-12, NO	Holán et al. (2003)
				↓IL-4, IL-10	
Morphine	*Vivo. and vitr.o*	BV-2, HEK 293T, PM, BMMs; C57BL/6 mice	P65, TRAF6, miR-124		Qiu et al. (2015)
M3G	*Vivo. and vitro.*	Sprague-Dawley rats, murine microglial cell line, BV-2	TLR4/MD2, CD11b	IL-1β	Lewis et al. (2010); Khabbazi et al. (2016)

When macrophages are stimulated by zymosan or LPS, opioids can promote the production of reactive oxygen species (ROS) intermediate and cytokines and reduce the expression of surface antigen presentation markers in peritoneal macrophages. Opioids promote the production of pro-inflammatory cytokines, including IL-6 and TNF-α, in the early stage. Secretion of IL-10 is then activated 1 day after the release of pro-inflammatory cytokines. All the antigen-presenting molecules (CD14, CD80, CD86, and MHC II) on the surface of macrophages are shown to be significantly inhibited by different opioids (morphine, fentanyl, or methadone) (Filipczak-Bryniarska et al., 2012). Limiroli et al. (2002) also found that morphine treatment can impair the production of cytokines by macrophages in mice, and the changes in cytokines IL-10 and IL-12 are altered by acute or chronic morphine treatment. Furthermore, Pacifici et al. (1995) found that NO_2_^−^ production by L1210-activated macrophages increased significantly immediately after injection of morphine, whereas NO_2_^−^ production was significantly inhibited by morphine after 24 h. Naloxone pretreatment completely antagonized the regulatory effects of morphine on NO_2_^−^ release. Alexander et al. demonstrated the effect of exogenous morphine on immune dysfunction in a mouse model of burns (Alexander et al., 2005). It was found that the combination of burn injury and morphine did not change the ability of macrophages to produce cytokines, but there was increased LPS-induced NO_2_^−^ production of spleen macrophages in morphine-treated mice.

The RelB factor plays an important role in the morphine regulation of macrophage cytokine production (Martucci et al., 2007). Morphine inhibits the expression of iNOS mediated by NF-κB in mice, which leads to the overactivation of cNOS and an increase in NO production. Morphine significantly decreased the production of pro-inflammatory cytokines IL-1, TNF-β, and IL-12 by animal macrophages, but had little effect on the anti-inflammatory cytokine IL-10. It has been suggested that RelB is an important target for morphine to regulate pro-inflammatory factors, but not anti-inflammatory factors. It should be noted that lack of RelB may alter the expression of cellular components downstream of the opioid receptor activation pathway and this factor may not be the direct target of opioid action.

In addition to morphine, some laboratories have explored and compared the effects of different opioids on macrophage cytokine production. Kozlowski et al. (2017) studied the effects of oxycodone and buprenorphine on the production of ROS intermediates and NO in peritoneal macrophages of mice *in vivo* and *in vitro*, and compared the effects of morphine, fentanyl, and methadone on macrophage immune function previously studied by Filipczak-Bryniarska et al. (2012). It was found that buprenorphine and oxycodone showed weaker immunomodulatory properties than morphine, which could prevent redundant inhibition of physiological immune defense. Some studies have found that the effect of heroin on macrophages is complex (Holán et al., 2003), for example, a study showed that within 2 h of heroin administration, the proliferation response to alloantigens and the production of IL-1β, IFN-γ, IL-12, and NO were significantly enhanced, while the production of anti-inflammatory cytokines IL-4 and IL-10 decreased.

There are several studies that specifically report the effects of opioids on macrophage polarization. Morphine may induce local macrophage phenotypic changes in the early stage of pain through the COX2/PGE2-dependent pathway (Godai et al., 2014). At the site of local morphine injection, pro-inflammatory F4/80 + iNOS + M1 macrophages decreased during pain formation, while F4/80 + CD206 + M2 macrophages increased in the early stage of wound healing. Buprenorphine, a synthetic opioid analgesic with MOP receptor activation and antagonism, has been found to have different regulatory effects on M1 and M2 macrophages (Sun et al., 2017). Buprenorphine inhibited the expression of many kinds of cytokine mRNAs and proteins in M1 macrophages and enhanced the expression of Ym1 and Fizz1 in M2 macrophages. In addition, buprenorphine did not affect the regulation of LPS on the cascade of NF-κB and MAPK in M1 macrophages but inhibited the expression of IRF5 and reduced the binding of DNA to IRF5. This suggests that buprenorphine can downregulate the IRF5 pathway and restrict the phenotype of M1 macrophages. Tramadol also has different regulatory effects on M1 and M2 macrophages. Tramadol regulates inflammation by inhibiting M1 macrophages, thereby inhibiting the killing process, and promoting M2 macrophage function, thereby promoting the healing process (Zhang et al., 2017). Tramadol significantly upregulated the expression of Arg1, Mrc1, Ym1, and Fizz1 in M2 macrophages. The STAT6 pathway may be the basis of tramadol’s effects because tramadol promotes the phosphorylation of STAT6, the Arg1 expression of STAT6, and the DNA binding of STAT6 in a dose-dependent manner.

In recent years, the polarization of macrophages has been a hot topic in research. The above research shows that opioids can affect the polarization of macrophages. The factors that affect the polarization direction of macrophages (M1 or M2) include the types of opioids and how they are used, the local environment of macrophages and physiological or pathological conditions. However, more basic and clinical research on the mechanism of opioids influencing macrophage polarization is needed in the future.

## Opioids Affect the Apoptosis of Macrophages

**FIGURE 1:**
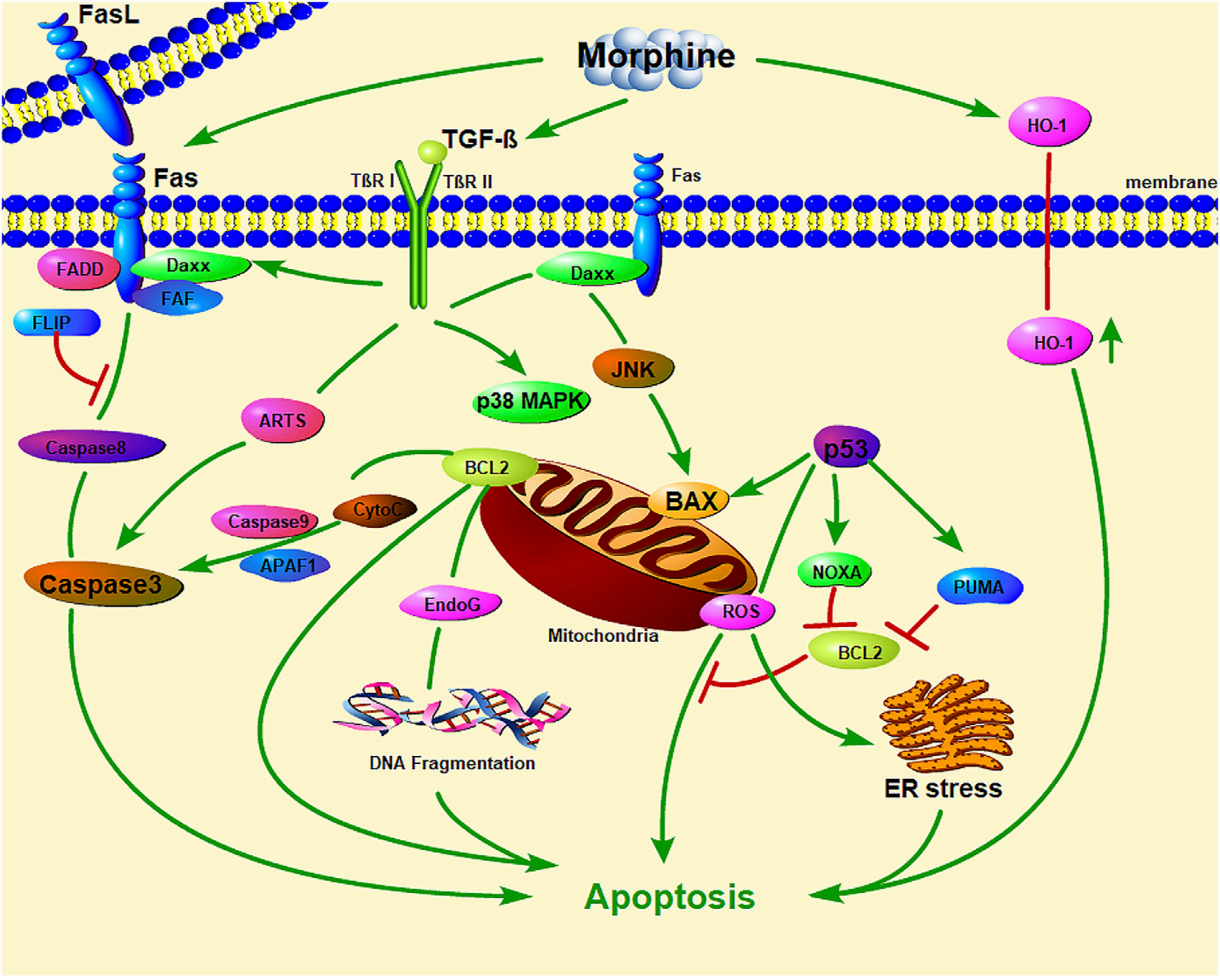
The important role of mitochondrial and endoplasmic reticulum stress in morphine-induced macrophage apoptosis. (Daxx: death domain-associated protein; FADD: Fas-associated with death domain protein; JNK: c-Jun N-terminal kinase; CytC: Cytochrome C; EndoG: Endonuclease G; PUMA: p53 upregulated modulator of apoptosis; ER stress: endoplasmic reticulum stress; HO-1: Heme oxygenase-1).

Studies showed that, morphine induced macrophage apoptosis in a dose-dependent manner (Table 3). Morphine-induced macrophage apoptosis is caused by morphine acting on opioid receptors through the p38 MAPK phosphorylation pathway. TGF-β and iNOS play important roles in morphine-induced downstream signal transduction (Singhal et al., 1998; Singhal et al., 2000; Singhal et al., 2002; Bhat et al., 2004), which seems to activate proteins involved in exogenous (Fas and FasL) and endogenous (p53 and Bax) cell death pathways (Figure 1). Morphine enhances iNOS mRNA expression in macrophages and induces macrophage apoptosis, which could be inhibited by iNOS inhibitors (L-NAME and L-NMMA), suggesting that morphine-induced macrophage apoptosis may be mediated by NO production. Morphine induces macrophage apoptosis through the accumulation of p53, while the period of morphine-induced apoptosis seems to be mediated by the accumulation of Bax and the activation of ICE-1 (Singhal et al., 1998). More interestingly, morphine-induced J774 cell apoptosis and Bax expression were inhibited by an anti-TGF-β antibody; therefore, morphine-induced J774 cell apoptosis may be mediated by the production of TGF-β (Singhal et al., 2000).

**TABLE 3:** Opioids affect the apoptosis of macrophages.

Opioids	Cell type/animal model	Mechanism or approach	Organelles involved	References
Morphine	J 774.16 cells	Induces oxidative stress; caspase-3 activation	Mitochondria, endoplasmic reticulum	Bhat et al. (2004)
Morphine	J774 cells	Through opiate receptors *via* P38 MAPK phosphorylation	Mitochondria, endoplasmic reticulum	Singhal et al. (2002)
		TGF-β and iNOS activate proteins involved in exogenous (Fas and FasL) and endogenous (p53 and Bax) cell death pathways		
Morphine	J774 cells	The generation of TGF-β	Mitochondria	Singhal et al. (2000)
	Murine peritoneal Mf			
Morphine	Sprague Dawley rats	Accumulation of p53 (the induction phase of apoptosis); accumulation of Bax and activation of ICE-1 (the effector phase)	Mitochondria	Singhal et al. (1998)
Morphine	FVB/N mice	Heme oxygenase-1 (HO-1)	Mitochondria	Patel et al. (2003)

Morphine also induces macrophage apoptosis in other ways. The apoptosis of macrophages is related to organelles such as mitochondria (Chen et al., 2020; Wang K. et al., 2020; Chen et al., 2021; Liu et al., 2021a; Liu et al., 2021b) (Figure 1). Mitochondria are not only the main sites for intracellular oxidative phosphorylation and the formation of ATP (Wei et al., 2022), but also the sites for the production of ROS (Forte et al., 2021; Wang et al., 2021). Oxidation and endoplasmic reticulum (ER) stress can accelerate the apoptosis of macrophages (Cominacini et al., 2015). It is worth noting that ROS inhibit the apoptosis of macrophages while mediating the apoptosis of other cells, especially tumor cells. This may be the basis for macrophages exerting tumor immunity. Bhat et al. found that this process involves the oxidative activation of NADPH with phospholipase D and calcium ions, leading to the production of superoxide (Bhat et al., 2004). Antioxidants have a protective effect on morphine-induced macrophage injury, which further confirms the role of mitochondrial oxidative stress in morphine-induced macrophage apoptosis. *in vivo* and *in vitro* experiments by Patel *et al.* showed that heme oxygenase-1 plays a role in morphine-induced macrophage migration and apoptosis (Patel et al., 2003).

**FIGURE 2:**
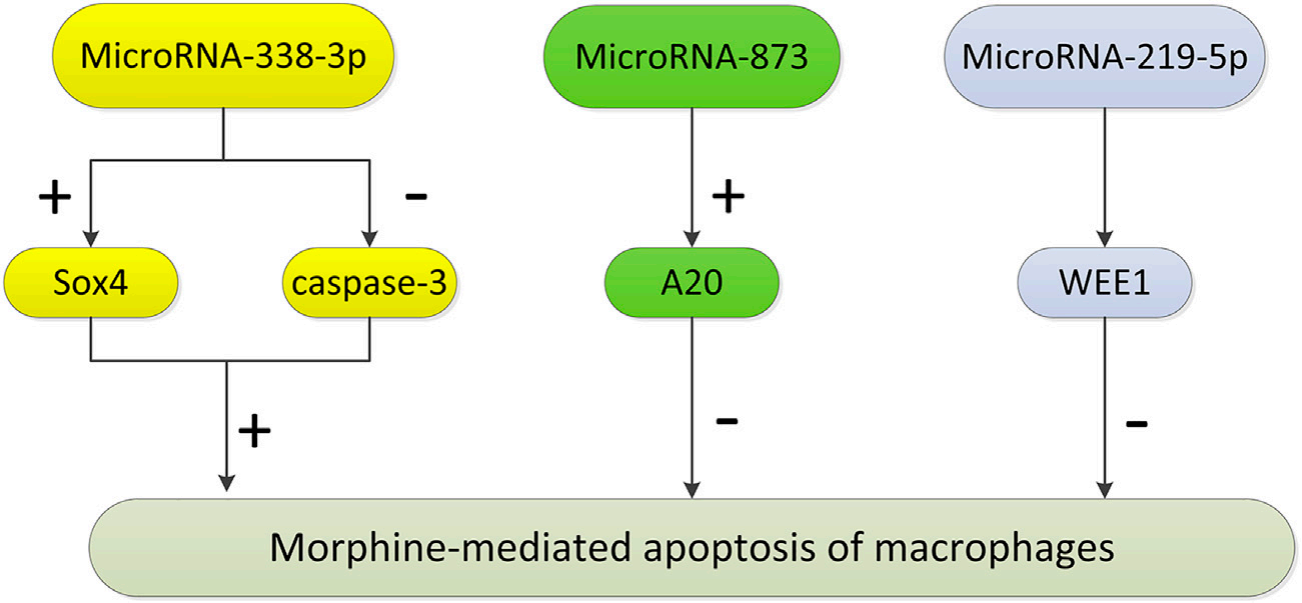
The pathways regulated by microRNAs in morphine-mediated apoptosis of macrophages. After morphine treatment, the expression level of miR-338-3p in peritoneal macrophages increased significantly, which decreased the expression level of Sox4, leading to the increase of caspase-3 expression level, and then promoting cell apoptosis. MicroRNA-873 inhibits morphine-induced macrophage apoptosis by increasing the expression of A20. MicroRNA-219-5p inhibits morphine-induced apoptosis by targeting WEE1, the key cell cycle regulator. (“+” represents promotion, and “−” represents inhibition.)

A variety of miRNAs control the molecular pathways involved in the regulation of the immune system and regulate many aspects of the immune response, including proliferation, differentiation, immune cell function, and intracellular signaling pathways. Some studies have found that these miRNAs play an important role in morphine-induced macrophage apoptosis (Figure 2). MiR-338-3p promotes cancer cell death by regulating specific signaling pathways or related genes (such as p38, mitogen-activated protein kinase, and AKT) during cancer treatment. Morphine may promote apoptosis by regulating the expression of miR-338-3p (Weng and Wang, 2016). After morphine treatment, the expression level of miR-338-3p in mouse peritoneal macrophages increased significantly, which decreased the expression level of Sox4, increased caspase-3 expression, and promoted cell apoptosis. MicroRNA-873 inhibits morphine-induced macrophage apoptosis by increasing A20 (Li et al., 2015). MicroRNA-219-5p inhibits morphine-induced apoptosis by targeting WEE1, a key cell cycle regulator (Lou et al., 2016).

## Effect of Exogenous Synthetic Opioids on Macrophages

In recent years, an increasing number of studies have explored the effects of synthetic opioids on immune function. For opioid receptor agonists, their activities on opioid receptors and their effects on immune cells can all differ. Selective MOP agonists are generally associated with immunosuppression, whereas DOP receptor-selective agonists are usually associated with immune enhancement (Gomez-Flores et al., 2001). KOP receptor agonists may play an important role in pulmonary inflammation by activating macrophages (Zeng et al., 2020). In this section, we will discuss the effect of exogenous synthetic opioids on macrophage function (Table 4).

**TABLE 4:** The effects of exogenous synthetic opioids on macrophage’s function.

Exogenous synthetic opioids	Animal models/cell types	Effects on macrophages	References
CGPM-9	Rat peritoneal macrophages	Inhibits the production of NO and TNF-α	Hicks et al. (2001)
SNC80	Rat peritoneal macrophages	Stimulates the production of NO and TNF-α	Gomez-Flores et al. (2001)
	Rat splenic macrophages		
DPDPE	Murine macrophage cell line (RAW 264.7)	Changes the dimer composition of NF-κB; slightly inhibit the production of MIP-2	Husted et al. (2005)
DADLE	Rat peritoneal macrophages; septic models	Inhibits the release of HMGB1, TNF-α, and IFN-γ	Tang et al. (2011)
U50488	Rat alveolar macrophage cell line (NR8383)	Anti-inflammatory effect on pulmonary macrophages	Zeng et al. (2020)
Salvinorin A	Mouse peritoneal macrophages	Moderate anti-inflammatory effects	Aviello et al. (2011); Zeng et al. (2020)
Ohmefentanyl	Rat peritoneal macrophages	Reduces the concentration of TNF-α and IL-1β; reduce phagocytic and bactericidal activity	Li et al. (2008)
Nalbuphine	Mouse contact allergic dermatitis model	Increases the production of M1 and IL-10	Inan et al. (2019)
MENK	Rat peritoneal macrophages	Regulates H_2_O_2_ release	Stanojević et al. (2008)
	Tumor-associated macrophages	Promotes the transformation from M1 to M2	Wang et al. (2018)

CGPM-9: 4-tyrosylamido-6-benzyl-1,2,3,4 tetrahydroquinoline; SNC80: 4-[alpha-(4-allyl-2,5-dimethyl-1-piperazinyl)-3-methoxybenzyl]-N,N-diethylbenzamide; DPDPE: (D2,5Pen)-enkephalin; DADLE: (D-Ala2, D-Leu5)-enkephalin; HMGB1: high-mobility group box 1 protein; U50488: trans-(±)3,4-dichloro-N-methyl-N-[2-(1-pyrrolidinyl)-cyclohexyl]-benzeneacetamide; MENK: methionine-enkephalin.

The non-peptide opioid, CGPM-9, activates the proliferation of thymocytes and inhibits the function of macrophages, including the production of NO and TNF-α, by acting on MOP receptors (Hicks et al., 2001). Intracerebroventricular injection of the non-peptide DOP receptor agonist, SNC80, did not alter some parameters of immune activity. However, at concentrations of 10^−7^ M and 10^−6^ M, SNC80 could significantly stimulate resident and LPS-stimulated peritoneal macrophages to produce TNF-α and NO (Gomez-Flores et al., 2001). The stimulation of the δ_2_-opioid receptor inhibits the activation of the p38MAPK pathway in macrophages, which is related to the decrease in TNF-α and MIP-2 production by macrophages. DPDPE, a specific δ_1_-opioid receptor agonist, can change the dimer composition of the transcription factor NF-κB and slightly inhibit the production of MIP-2 (Husted et al., 2005). In addition, DADLE can inhibit the release of HMGB1 from macrophages induced by LPS, TNF-α, and IFN-γ. DADLE may protect rats with sepsis by reducing serum HMGB1 levels (Tang et al., 2011). U50488, a selective KOP agonist, had a strong anti-inflammatory effect on pulmonary macrophages within one to 2 hours after LPS-stimulated inflammatory response *in vitro* (Zeng et al., 2020). Salvinorin A (SA) is an effective KOP agonist, which can exert an intense effect on macrophages through KOP and cannabinoid CB1 receptors and shows moderate anti-inflammatory effects *in vivo* (Aviello et al., 2011; Zeng et al., 2020).

*In vitro*, ohmefentanyl inhibited the immunosuppressive function of rat peritoneal macrophages, including reducing the concentration of TNF-α and IL-1β, as well as inhibiting phagocytic and bactericidal activity (Li et al., 2008). Nalbuphine is a KOP receptor agonist and a MOP receptor antagonist, which can reduce pruritus and increase the production of M1 macrophages and IL-10 in a mouse model of contact dermatitis (Inan et al., 2019). The regulation of methionine-enkephalin (MENK) on the release of H_2_O_2_ from rat peritoneal macrophages involves different types of opioid receptors. The enhancement of H_2_O_2_ release induced by MENK is mediated by the functional interaction of δ_1_ or δ_2_ opioid receptor subtypes or MOP-KOP receptors, while the inhibition of MENK induced release involves the functional interaction between δ_1_ and MOP, δ_2_ and MOP or δ_1_ and KOP receptors (Stanojević et al., 2008). MENK may cause macrophages in tumors to change from the M1 phenotype to the M2 phenotype and induce apoptosis by blocking the OGFr/PI3K/AKT/mTOR signalling pathway (Wang et al., 2018). Recently, Tian et al. (2020) found that MENK can inhibit influenza A virus infection and this antiviral effect is related to the promotion of opioid receptors (MOP) and the activation of NF-κB p65 to induce an antiviral state.

## The Regulation of Endogenous Opioid Peptides on Macrophages’ Function

In addition to exogenous synthetic opioids, many immune cells also secrete endogenous opioid peptides, including enkephalins, endorphins, dynorphins, FQ nociceptin/orphanin peptide, and endomorphins 1 and 2 (Gein and Baeva, 2011). They also work through opioid receptors and can bind to opioid receptors on immune cells to regulate immune function. In this section, we review the role of endogenous opioid peptides in regulating the key functions of macrophages (Table 5).

**TABLE 5:** The effects of endogenous opioid peptides on macrophage’s function.

Endogenous opioid peptides	Animal models/cell types	Effects on macrophages	References
Endomorphin	Rat peritoneal macrophages	Enhances the adhesion of macrophages and the expression of the adhesion molecule Mac-1; inhibits the chemotaxis and superoxide anion production of macrophages; inhibits the production of TNF-α, IL-10, and IL-12	Inui et al. (2002)
	Murine macrophage cell line, J774; mice peritoneal macrophages	Activates NOS2 activity, downregulate NOS2 gene expression, and inhibit the release of NO	Sarić et al. (2007); Balog et al. (2010)
	Human lipid-laden macrophages	Regulates the release of cytokines by human lipid macrophages by downregulating CD36	Chiurchiù et al. (2011)
β-endorphin	THP-1 monocyte-derived macrophages	Increases oxLDL uptake by macrophages and promote oxLDL-induced macrophages to form foam cells; transforms the macrophage phenotype into pro-inflammatory M1 through NF-κB phosphorylation; increases macrophage migration and apoptosis	Okano et al. (2020)
Dynorphin A	Murine IC-21 macrophages	Enhances Mac-1-mediated phagocytosis of macrophages	Podolnikova et al. (2015)

NOS2: Nitric oxide synthase 2; oxLDL: oxidized low-density lipoprote.

Endomorphin is a newly discovered MOP receptor-selective immunoreactive opioid peptide. Endomorphin-1 is mainly distributed in the brain, whereas endomorphin-2 is widely distributed in the spinal cord. Endomorphin-1 can enhance the adhesion of macrophages and the expression of the adhesion molecule Mac-1 but does not affect the phagocytosis by macrophages of *Escherichia coli.* In addition, endomorphin-1 not only inhibits the chemotaxis and superoxide anion production of macrophages, but also inhibits the production of TNF-α, IL-10, and IL-12 by macrophages stimulated by LPS (Inui et al., 2002). Endomorphin-1 can activate NOS2 activity, downregulate NOS2 gene expression, and inhibit the release of NO, which seems to be mediated by the MOP-opioid receptor (Sarić et al., 2007; Balog et al., 2010). It is worth noting that endomorphin-1 can inhibit lipid accumulation and regulate the release of cytokines by human lipid macrophages by downregulating CD36, suggesting the potential for new treatments of anti-atherosclerosis based on endomorphin (Chiurchiù et al., 2011).

β-endorphin is an endogenous opioid peptide that can play various roles in the whole body. β-endorphin, as the main agonist of MOP receptors, can be found in the brain and immune system cells. β-endorphin can increase oxidized LDL (oxLDL) uptake by macrophages and promote oxLDL-induced macrophages to form foam cells. This effect confirms that there is a close relationship between endogenous opioids and the ER metabolism of macrophages. In the process of mononuclear macrophage differentiation, β-endorphin also significantly transformed the macrophage phenotype into pro-inflammatory M1, rather than anti-inflammatory M2, through NF-κB phosphorylation. Furthermore, β-endorphin was also associated with c-Jun-N terminal kinase, p38, and NF-κB phosphorylation, which increases macrophage migration and apoptosis (Okano et al., 2020).

Integrin Mac-1 is a multi-ligand receptor that mediates a variety of monocyte/macrophage responses in the immune-inflammatory response. Dynorphin A can induce strong migration of leukocytes expressing Mac-1 and enhance Mac-1-mediated phagocytosis of latex beads by mouse IC-21 macrophages (Podolnikova et al., 2015).

## Opioids, Macrophages, and HIV Infection

**FIGURE 3:**
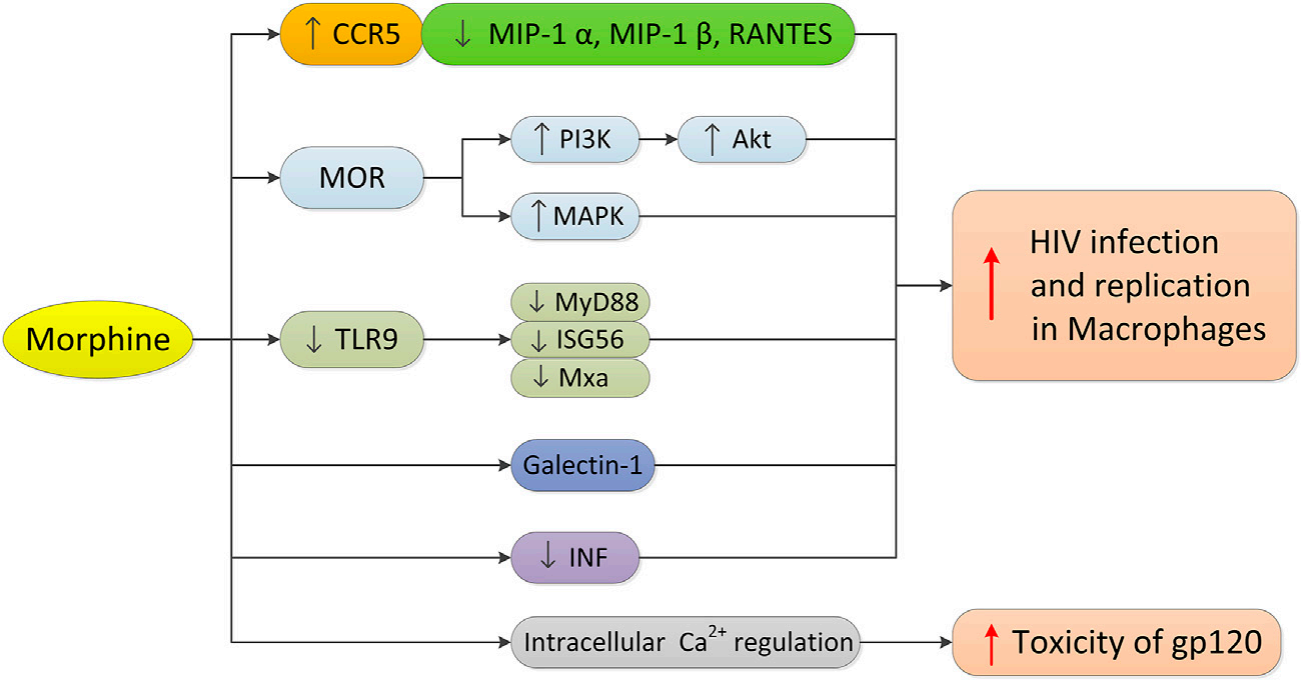
Morphine’s mechanism in regulating HIV infection in macrophages. Morphine promotes the infection and replication of HIV in macrophages as follows: 1) upregulating the expression of CCR5 and downregulating the expression of CCR5 competitive ligand in macrophages; 2) activating MOR, PI3K/Akt and MAPK signaling pathways; 3) inhibiting the TLR9 pathway and down-regulating the expression of MyD88, ISG56 and Mxa; 4) increasing the expression of Galectin-1; and 5) significantly inhibiting the interferon signaling pathway and interferon-induced gene expression, and destroying the inherent antiviral mechanism of macrophages. In addition, morphine can enhance the cytotoxicity of HIV-1 virus protein gp120 through intracellular calcium regulation.

Macrophages play an important role in all stages of HIV infection. They are not only the main target cells and repositories of HIV, but also the transmitters of HIV to CD4+T cells. CC chemokine receptor 5 (CCR5) is necessary for entry of macrophages by R5 HIV. Morphine may promote the entry of HIV into cells by increasing the expression of CCR5, thus promoting HIV infection and virus replication in monocytes/macrophages (Guo et al., 2002; Li et al., 2003) (Figure 3). The MOP receptor agonist, methadone, can also increase HIV infection in adult macrophages, which is also related to the upregulated expression of CCR5 (Li et al., 2002). In contrast, morphine can downregulate the production or expression of the CCR5 β chemokine ligand (MIP-1 α, MIP-1 β, or RANTES) in human macrophages. Since CCR5 receptor interaction promotes HIV infection and replication, morphine may increase HIV entry into macrophages by downregulating the expression of competitive CCR5 receptor ligands. It is worth noting that there is an interaction between the opioid receptor and chemokine receptor CCR5 on macrophages, and the oligomerization of the two receptors on the cell membrane may regulate receptor function (Suzuki et al., 2002). The selective KOP ligand, U50488, can inhibit HIV-1 expression in acutely infected h-MDMS, indicating that the KOP ligand may have therapeutic potential in the treatment of AIDS (Chao et al., 2001).

HIV affects microglia and astrocytes, resulting in neurodegenerative changes. In HIV-infected opioid abusers, central nervous system inflammation may increase even when HIV infection is under control (Murphy et al., 2019). Opioids can enhance the cytotoxicity of the HIV-1 virus protein gp120 through an intracellular calcium regulation mechanism, making it an important cellular target for HIV-opioid interaction (Mahajan et al., 2005). Opioids not only directly affect astrocytes and macrophages or microglia that express MOP (Hauser et al., 2005; Bruce-Keller et al., 2008), but also regulate inflammation and disrupt the interaction between normal immune cells, including macrophages and lymphocytes. The neural pathways involved in opioid enhancement of HIV-induced inflammation and cell death appear to involve the activation of MOP and downstream effects through PI3K/Akt and/or MAPK signal transduction (Hauser et al., 2005). Recent studies by Liao et al. (2017) have shown that combined with HIV-1 infection, morphine reduces the expression of MyD88, ISG56, and Mxa in macrophages by inhibiting the TLR9 pathway, which in turn promotes the replication of HIV-1 in macrophages.

Many studies have shown that opioids can promote HIV infection by regulating the expression of various factors in human macrophages. Morphine can enhance the effect of HIV gp160 protein on macrophage apoptosis, iNOS expression, and NO production (Kapasi et al., 2004). Chronic morphine exposure to HIV-infected h-MDMs can lead to significant changes in the secretion of IL-6 and monocyte chemoattractant protein-2 (MCP-2) (Dave, 2012). Morphine promotes the secretion of h-MDMs infected with HIV and inactivates the secretion of MCP-2 by IL-6, which has a potential additive effect. In addition, the increased expression of Galectin-1 induced by morphine may also regulate HIV-1 infection and increase the infection of HIV-1 (Reynolds et al., 2012). H-MDMs treated with morphine and methadone can significantly inhibit the interferon signaling pathway and interferon-induced gene expression (Wang et al., 2012; Wang MR. et al., 2020), thus damaging the inherent antiviral mechanism in macrophages and increasing the susceptibility of cells to HIV infection.

Differentially expressed miRNAs (hsa-miR-15b and 181Mub) may play a potential role in regulating morphine-induced inflammation and oxidative stress in h-MDMS, leading to the expansion of the central nervous system reservoir of HIV-1 and the progression of AIDS (Dave and Khalili, 2010). Heroin and methadone can inhibit microRNA restriction (miRNA-28, -125b, -150, and -382) and enhance HIV infection and replication in macrophages (Wang et al., 2015; Wang MR. et al., 2020). Sudden or slow withdrawal of morphine can inhibit the expression of many HIV inhibitors in macrophages, including APOBE3G/F, SAMHD1, MX2, and HIV-restricted microRNA (miR-28, miR-125b, and miR-150), and further enhance the sensitivity of macrophages to HIV infection (Wang et al., 2019).

## Opioids, Macrophages, and Tumors

Some studies have shown that TAMs play an important role in tumor invasion and metastasis, and the role of TAMs in tumors is almost the same as that of M2 macrophages (Rolny et al., 2011). This view has been confirmed by TAM and M2 macrophage expression profiles (Porta et al., 2009). The conversion of M1 macrophages to M2 in tumors has also become one of the main directions of current research. Many studies have shown that opioids can combine with opioid receptors to regulate immunity or other pathways that affect the occurrence, development, and prognosis of tumors (Fujioka et al., 2011; Kuzumaki et al., 2012; Long et al., 2016).

Morphine can regulate tumor invasiveness by regulating the production of macrophage proteases and M2 polarization in the tumor microenvironment (Khabbazi et al., 2015). IL-4 leads to the production of MMP-9 and increased expression of the M2 markers Arg-1 and MRC-1. Morphine inhibits IL-4-induced increase of MMP-9 and selective activation of macrophages in a reversible manner with naloxone and methylnaltrexone. When macrophage cell line RAW264.7 was subjected to paracrine activation by 4T1 breast cancer cells, the expression of MMP-9 and Arg-1 increased, and this effect was blocked by morphine through an opioid receptor-mediated mechanism. Morphine further reduced the invasion of 4T1 breast cancer cells co-cultured with RAW264.7.

There is also evidence that opioid receptor agonists or antagonists affect tumor growth by regulating macrophage function. The opioid receptor agonist leu-enkephalin has an anti-survival effect in renal clear cell carcinoma, mainly through Th2 immunity and the NRF2-dependent macrophage network (Scarpa et al., 2020). Low-dose naltrexone (LDN) reduces tumor size by increasing the level of M1-like macrophages and activating the Bax/Bcl-2/caspase-3/PARP signaling pathway to induce apoptosis (Ma et al., 2020). In addition, LDN indirectly reduced the number of TAMs (mainly M2) and decreased the expression of anti-inflammatory factor IL-10 in the serum of nude mice, indicating that LDN may be a potential treatment for cervical cancer (Liu et al., 2020).

In short, the effects of opioids on macrophage subtypes may further affect the occurrence and development of tumors. Research in this direction may provide a new strategy for the use of opioids, such as morphine, and the prognosis and treatment of tumors.

## Opioids and Organelles in Macrophages: A Valuable Research Direction

Organelles are generally regarded as micro-structures or micro-organs with certain morphology and functions dispersed throughout the cytoplasm of cells. Key organelles in macrophages include mitochondria, endoplasmic reticulum, Golgi apparatus, ribosomes, lysosomes, and centrosomes. They constitute the basic structure of macrophages, which enable cells to work and operate normally and enable macrophages to play a role in human physiological and pathological conditions. In view of the important role of mitochondria and endoplasmic reticulum in the metabolism and function of macrophages, we mainly discuss these two organelles here.

There is growing evidence that mitochondria are central regulators of metabolic reprogramming and control the activation and function of immune cells (El Kasmi and Stenmark, 2015; Corrêa-da-Silva et al., 2018; Liu and Ho, 2018; Ramond et al., 2019). Macrophages that differentiate into M1 can increase glucose uptake and glycolysis (Rodríguez-Prados et al., 2010). This is associated with the production of HIF1α. HIF1 induces the production of the pro-inflammatory factor IL-1β and the upregulation of several enzymes associated with glycolysis (Tannahill et al., 2013). The expression of carbohydrate kinase-like protein, which inhibits the pentose phosphate pathway, is down-regulated in M1 macrophages, leads to an increased pentose phosphate pathway (Haschemi et al., 2012). The pentose phosphate pathway produces nucleotides and NADPH, the latter of which is important for mitochondrial ROS production (Haschemi et al., 2012). M1 macrophages also increase glutamine metabolism, and glutamine is used as α-ketoglutaric acid in the TCA cycle after its decomposition (Tannahill et al., 2013). This stimulates the accumulation of succinic acid (Tannahill et al., 2013). As mentioned earlier, the TCA cycle of M1 macrophages is impaired with two defective steps (Tannahill et al., 2013; Jha et al., 2015). The first defective step results in the accumulation of citric acid in the cytoplasm of cells (Newsholme et al., 1986). Citric acid can then be used to synthesize lipids (prostaglandins), NO, or ROS (Infantino et al., 2011), which are important for the function of M1 macrophages. Another defective step in the TCA cycle results in the accumulation of succinic acid (Tannahill et al., 2013), which stabilizes HIF1α. With the increase of glycolysis, the coordinated rearrangement of the TCA cycle, and the decrease of mitochondrial oxidative phosphorylation, the production of intermediates (such as succinic acid, citric acid) and ROS/NO is promoted. The accumulation of these substances plays a specific role in enhancing the ability of macrophages to initiate an inflammatory response and participate in paracrine signals (El Kasmi and Stenmark, 2015). In addition, like the role of mitochondrial nucleotides in inducing inflammation (Kuck et al., 2015), the regulation of macrophage plasticity, by the reprogramming of mitochondrial metabolism, has become another important feature of mitochondria. More interestingly, the increase of aerobic glycolysis is a key prerequisite for maintaining mitochondrial membrane potential, thus preventing macrophage apoptosis. Compared with M1, M2 macrophages do not increase glycolysis (Rodríguez-Prados et al., 2010). The metabolism of M2 macrophages depends on the TCA cycle and oxidative phosphorylation (mainly β-oxidation) (Vats et al., 2006; Huang et al., 2014). Enhanced β-oxidation was associated with increased TCA, increased respiratory capacity, and thus with an increased ability to produce ATP through oxidative phosphorylation. This may be important for the physiological function of M2 macrophages.

The dysfunction of organelles caused by various factors often leads to metabolic disorders and functional disorders of macrophages. The endoplasmic reticulum (ER) is a complex cytoplasmic membrane structure involved in protein synthesis, folding and modification, lipid synthesis and transport, and intracellular calcium balance regulation (Sukhorukov et al., 2020). ER stress severely interferes with ER function, Ca^2+^ signaling, and protein synthesis, and is associated with a variety of pathophysiologies, such as macrophage apoptosis (Scull and Tabas, 2011; Cominacini et al., 2015), efferocytosis (Linton et al., 2016), foam cell formation (Han and Kaufman, 2016), and inflammation. ER stress can activate or accelerate apoptosis of macrophages through a variety of pathways, such as excessive accumulation of lipids (free cholesterol, sterols, and oxLDL), inflammatory pathways (acceleration of INF-γ), oxHDL binding to TLR4, and increased Ca^2+^ concentration in the ER leading to mitochondrial uptake of calcium ions. ER stress regulates lipid metabolism in macrophages by stimulating cholesterol uptake, inhibiting cholesterol outflow, and regulating the expression of cholesterol membrane transporters, resulting in lipid accumulation and subsequent differentiation into foam cells in macrophages (Sukhorukov et al., 2020). Addressing how to mitigate metabolic disorders by controlling ER stress-mediated macrophage plasticity is crucial for the progression of atherosclerosis. In addition, ER stress may be involved in cellular inflammation through NF-κB, activated protein-1 and JNK signaling pathways, and ROS production (Sukhorukov et al., 2020). A link between cholesterol accumulation, ER stress, and the pro-inflammatory response has been established, but the complex relationships between the processes involved still need to be described in detail. Some studies have also emphasized the key role of mitochondria in the response of macrophages to bacterial pathogens (Ramond et al., 2019). Mitochondria can meet the energy needs of cells and maintain the phagocytosis of macrophages in the lytic stage. Mitochondrial disorders may be a cause of susceptibility to bacterial infection.

Mitochondria and other organelles play an irreplaceable role in the cellular metabolism and immune response of macrophages. It has already been mentioned that mitochondrial and ER stress is extremely important in morphine-induced macrophage apoptosis (Figure 1). However, there are limited studies on the effects of other opioid drugs on the functions of various organelles of macrophages. Exploring how opioids control the flexibility of macrophage metabolic programs and influence the connections between macrophage metabolism and transcriptional networks during inflammatory activation and anti-inflammatory processes is crucial to better understand the effects of opioids on macrophage plasticity and macrophage biology. Whether opioids can change macrophages from a pro-inflammatory phenotype to a less inflammatory phenotype or even repair macrophage phenotype, remains to be further studied.

## Conclusion

Opioids are one of the most effective painkillers that can be used to treat pain in the clinic. It has been confirmed that opioids have potential effects on the functioning of the immune system. Many studies have explored the relationship between macrophages and different opioids *in vitro*, *in vivo*, and in epidemiological and clinical studies in different patient groups. Opioids mediate the effects on macrophages through direct and indirect mechanisms. The binding with various opioid receptors on the surface of macrophages is a direct actional pathway, affecting the migration and phagocytic activity of macrophages. Indirect effects include the production of opioid receptors and endogenous opioid peptides in the central nervous system. Because macrophages provide antigens for lymphocytes, the effects of opioids on macrophage function may change host immune defense and changes in macrophage function may also affect the immune response. Different types of opioids, different doses, and administration methods have different regulatory effects on macrophages and their subtypes. The single and combined effects of opioids may impair the functioning of macrophages in the host defense system. Morphine induced macrophage apoptosis in a dose-dependent manner, indicating that the impairment of macrophage function induced by morphine may be indirectly caused by morphine-induced macrophage apoptosis. A number of studies have shown that opioids can regulate macrophages to promote HIV infection and the progression of AIDS in a variety of ways. Exploring suitable opioid receptor ligands may be one of the strategies for the treatment of AIDS. Opioids can combine with opioid receptors to regulate immunity or other pathways that affect the occurrence, development, and prognosis of tumors. Therefore, how opioids affect tumor progression through macrophage subtypes is worth exploring.

Although there have been some studies on the effects of opioids on macrophages, the mechanism is still not clear, and more prospective studies are needed. In addition, the study on the effects of commonly used opioids, such as fentanyl and sufentanil, on macrophages during the perioperative period can provide suggestions for the guidance of perioperative drug use, but some studies are limited due to the particularity of narcotic drugs. The study of the possible correlation between the use of opioids and macrophage effects will help to provide a theoretical basis for the better application of opioids in clinical pain treatment and tumor outcome and treatment.

## Abbreviations

AIDS, Acquired Immune Deficiency Syndrome; cAMP, Cyclic adenosine monophosphate; DOP, δ-opioid; ER, endoplasmic reticulum; GPCRs, G protein-coupled receptors; HDM, high dose of morphine; HIF1α, hypoxia-inducible factor 1α; HIV, human immunodeficiency virus; h-MDMs, human monocyte-derived macrophages; IRF5, Interferon regulatory factor 5; KOP, κ-opioid; LDN, Low-dose naltrexone.; MAPK, mitogen-activated protein kinase; MCP, monocyte chemoattractant protein; MMP-9, matrix metallopeptidase 9; MOP, μ-opioid; NC-IUPHAR, the Committee on Receptor Nomenclature and Drug Classification; NO, nitric oxide; NOP, nociceptin receptor; NOS2, nitric oxide synthase 2; oxLDL, oxidized low-density lipoprote; ROS, reactive oxygen species; RT-PCR, Reverse Transcription-Polymerase Chain Reaction; TAMs, Tumor-associated macrophages; TCA, tricarboxylic acid
